# Quality of Nursing Work Life among Nurses in Saudi Arabia: A Descriptive Cross-Sectional Study

**DOI:** 10.3390/nursrep12040097

**Published:** 2022-12-16

**Authors:** Abbas Al Mutair, Mohammed I. Al Bazroun, Eman M. Almusalami, Faiza Aljarameez, Amal I. Alhasawi, Fatimah Alahmed, Chandni Saha, Hanan F. Alharbi, Gasmelseed Y. Ahmed

**Affiliations:** 1 Research Center, Almoosa Specialist Hospital, Al-Ahsa 36342, Saudi Arabia; 2 School of Nursing, University of Wollongong, Wollongong 2522, Australia; 3 Almoosa College of Health Sciences, Al-Ahsa 36342, Saudi Arabia; 4 Nursing Department, Prince Sultan Military College, Dhahran 34313, Saudi Arabia; 5 Nursing Department, Qatif Central Hospital, Qatif 32654, Saudi Arabia; 6 King Saud bin Abdulaziz for Health Sciences, King Abdullah International Medical Research Center, Ministry of the National Guard Health Affairs, Al-Ahsa 36361, Saudi Arabia; 7 Maternity and Child Health Nursing Department, College of Nursing, Princess Nourah Bint Abdulrahman University, Riyadh 84428, Saudi Arabia; 8 College of Medicine and Health Sciences, Almanagil University, Al-Jazirah 21121, Sudan

**Keywords:** quality, nurse, life, work, QNWL

## Abstract

**Background:** Quality of working life (QWL) is a multidimensional concept that describes an employee’s satisfaction with several work life elements. Quality of nurse working life is considered as a stepping stone for health services improvement, as it affects job satisfaction which, in turn, affects the performance of nurses. Understanding and investigating the nurses’ quality of work life in Saudi Arabia is needed for improvement actions. **Objectives:** This study aimed to examine the quality of nursing work life (QNWL) among nurses working in Saudi Arabia and to determine the association between demographic variables and quality of work life among nurses. **Methods:** It was a cross-sectional design using Brooks’ quality of nursing work life survey. It was distributed among nurses over the kingdom of Saudi Arabia. **Results:** There were 860 nurses participating in the study. The mean total score for the participants was 174.5+/− 30.3, indicating moderate to high QNWL. The highest score achieved by the nurses was for the work world context (4.29) while the lowest score was for work design dimension (3.92). The study revealed that nationality, income, and shift duration, having a dependent person, and having family accompany the nurse as significant factors affecting the quality of work life among the nurses. **Conclusion:** A novel contribution of the current study was that the demographic characteristics of the participants, including nationality, income, having family accompany the nurse, having an independent child, or spouse or parents, and shift duration, tended to have a statistically significant correlation with QNWL. The comprehensive results of this study have practical implications whereby authority bodies can create regulatory plans for enhancing satisfaction and performance over the sole utilization of job satisfaction measurements and can thereby improve nurses’ retention and turnover rates.

## 1. Introduction

Quality of working life (QWL) is a multidimensional concept that describes an employee’s satisfaction with several work life elements. These elements include factors such as job security, job satisfaction, work environment, job affairs, career prospects, nature of job, and work–life balance [1]. In the nursing literature, Brooks defined the quality of work life as “the degree to which registered nurses are able to satisfy important personal needs through their experiences in their work organization while achieving the organization’s goals” [2]. The notion of worker contentment is vital, as a worker will feel comfortable when they are recognized, acknowledged, needed, and respected [3]. The results of existing studies on the QWL of nurses indicated that nurses complained of heavy workloads, poor staffing, lack of autonomy, shared decision-making, and performing non-nursing tasks [2,4].

Quality of nurse working life is considered as stepping stone for health services improvement [5]. Quality of nursing work life affects job satisfaction, which, in turn, affects the performance of nurses [6,7]. Nurses’ satisfaction with QWL is associated with positive outcomes, such as improving quality of provided care, improving productivity and performance, enhancing retention rates, and reducing turnover [8]. In contrast, nurses’ dissatisfaction with QWL may lead to serious consequences that affects nurse’s personal life, which in turn may affect the quality of nursing care and threaten safety of patients [9]. Therefore, improving quality of nursing work life is critical for better nursing services [10]. There are number of identified factors that have an impact on the QWL of nurses, such as lack of work–life balance, work overload, and working environment [2,4,11]. Numerous research studies among nurses in different settings and different countries of the world has been conducted to measure nurses’ working life quality, and to assess factors affecting the working life quality of the nurses. Results from different studies showed that most nurses were not satisfied with their working life quality, and there were some major issues with the nurses’ quality of working life that need to be addressed in order to improve the nursing profession.

Based on the Saudi Ministry of Health statistics, even with the ongoing Saudization plan, the health care system still relies heavily on non-Saudi nurses [12]. In 2018, out of a total of 125,379 nurses, only 12,607 (approximately 10 percent), were Saudi. The ratio of nurses to the population is only about 5.5 + non-Saudi nurses per 1000 population and 2.1 per 1000 population Saudi nurses, which is lower than the United States, United Kingdom, and Canada. In addition, nursing turnover rates are estimated to be around 20 percent, higher the rate of countries such as the United Kingdom [13].

Studies in Saudi Arabia reported overall dissatisfaction among nurses with QWL. Almalki et al., 2012 reported significant job dissatisfaction amongst primary care nurses in one region of Saudi and a high turnover rate of 40% [14]. Furthermore, Alharbi et al., 2019 showed that nurses in Madinah region of Saudi Arabia had a moderate QNWL [15]. The diverse nature of the nursing workforce in Saudi Arabia generates multiple challenges for both Saudi and non-Saudi nurses. Cultural and language barriers as well as the reported dissatisfaction aggravate the problem of nurse shortage and turnover. Therefore, understanding and investigating the nurses’ quality of work life in Saudi Arabia is required. Thus, this study aimed to examine the quality of work life among nurses in different regions of Saudi Arabia and to determine the relationship of QWL with selected socio-demographic variables.

## 2. Methods

### 2.1. Study Design

The study was a descriptive cross-sectional design through an online questionnaire including nurses working in different regions of Saudi Arabia. The data were collected from November 2021 to January 2022. The study used a non-probability convenience sample. Ethical approval was obtained from Qatif Central Hospital ethical committee (IRB log number: QCH-SRECO 294/2021), and the study was conducted in accordance with the Declaration of Helsinki. The inclusion criteria for sample selection were all inpatient bedside staff nurses with non-leader positions. Nurses who worked as educators and out-patient nurses were not eligible to participate in the study. The manuscript reporting adhered to the STROBE guideline in the current study [16].

The sample size was determined by using the following power analysis formula $N=(Z_{\alpha }{)}^{2}p\left(1-p\right)/E^{2}$, leading to 780 participants needed for the survey. A total of *n* = 860 nurses fully responded to Brooks’ quality of nursing work life survey in Saudi Arabia in 2022, giving a response rate of 87%. To ensure the representativeness of the sample, a random selection of nurses with diverse biodemographics from multiple health settings and different work units was implemented.

### 2.2. Data Collection Tool and Validation

Brooks’ quality of nursing work life (QNWL) questionnaire was used. This questionnaire was developed by Brooks and Anderson (2005) to measure the QWL among nurses [5]. It is a self-report questionnaire with 42 items divided into four subscales: work life/home life (7 items), work design (10 items), work context (20 items), and work world (5 items). The work life/home life dimension is defined as the interface between the professional and personal life of the nurse. The work design dimension relates to the nature and composition of the actual work that nurses perform. The work context dimension includes the physical nature of the practice setting that have an impact on both nurses and patient systems. The work world dimension includes the broad societal influences and changes in relation to nursing practice. The instrument has a 6-point scale for each item, 1 indicating “strongly disagree” up to 6 indicating “strongly agree”. Brooks set up a cut-off point for the total score to indicate the levels of QNWL as follows: low (42–112), moderate (113–182), and high (183–252) [17]. Thus, a high overall score indicated a high QNWL [5]. Brooks et al., 2007 reported that the survey was pilot tested with a convenience sample of registered nurses who closely resembled the registered nurses in the sample [4]. Test-retest reliability was 0.90. Cronbach’s alphas for the dimensions were: work life/home life 0.56, work design 0.58, work context 0.88, and work world 0.60. The QNWL scale was reported to have acceptable construct validity (Cronbach’s alpha = 0.89) and test-retest reliability (intraclass correlation coefficient = 0.9) [18]. Brooks reported that the Brooks QNWL had a high internal consistency coefficient of (Cronbach α = 0.89) [17]. Brooks and Anderson (2004) reported a high test-retest reliability among 53 registered nurses over a 14-day interval between testing for the total Brooks QNWL score (r = 0.90, *p* < 0.001) [2]. Lee et al., 2014 provided evidence of discriminant validity with a significant positive Pearson correlation (r = 0.72, *p* < 0.01) between Brooks’ QNWL and the practice environment scale [19]. Additionally, there was evidence of concurrent validity with a significant weak negative correlation (r = −0.22, *p* < 0.01) between Brooks’ QNWL and Beck’s depression inventory in an Arab nursing sample (*n* = 508). The internal consistency reliabilities of the total Brooks QNWL scores were 0.89 [20]. Using this scale will assuredly generate reliable results and thus it was used in the present study to measure quality of nursing work life.

### 2.3. Statistical Analysis

After obtaining nurses’ responses, data were cleaned and transported to SPSS version 25 for statistical analysis. Descriptive and inferential statistics were conducted with a pre-set *p*-value of ≤ 0.05 as the accepted significance level for all statistical tests. Continuous variables were expressed and reported as mean and standard deviations. Categorical variables were presented as counts and proportions (%). Univariate analysis for comparison of outcome variables in relation to different categorical predictor variables was reported using chi-square or Fisher’s test. Data are available on request from the author.

## 3. Results

There were 860 nurses participating in the study. Out of the total number of respondents, 92.8% were female, and 61.3% of the study participants were Saudi. The mean age was 33.2 ± 6.1.

Around three-quarters of the participants were married and their families accompanied them. Half of the study participants received a salary of more than SAR 10,000 while the second half had a salary of less than SAR 10,000. Around 90% of the participated nurses were working for the government health care facilities. Only 17% of the study participants had chronic medical issues. About 44% of the participated nurses were responsible for elderly parents or a spouse, and 33.6% of the them were responsible for the care of a special needs child. The results of demographic data are presented in Table 1.

The total score of the QNWL was 174.5 +/− 30.3 which was obtained by adding the four subscales’ scores and dividing them by the number of items in the scale, which is four. Around 57.6% of the participating nurses had a moderate QNWL in which their total score was between 113 and 182. While 41.4% of the study participants had a high QNWL, only 1% had a low QNWL. Looking into the subscale, work context scored the most (4.29) while work design scored the least (3.62) (see Table 2).

Studying the association between different QNWL categories and participants’ demographic characteristics showed that a *p*-value was less than 0.05 with different nationality, income rang, whether family accompanied the nurse or not, working hours per shift, and whether the nurse was responsible for parents, special needs spouse, or child. Among nurses with low QNWL, eight of them were Saudi while only one was non-Saudi. For high QNWL, there was only 3% difference in the number of nurses between Saudi and non-Saudi. Furthermore, the majority of patients with high QNWL score (63.8%) had their families accompany them in Saudi; *p*-value was 0.001. For income difference, around 41% of the nurses with a high QNWL had a salary of more than 10,000 SAR. Comparing the working hours per shift, 33.1% of nurses with a moderate QNWL were working 9.5 h per shift. Additionally, 61% of the participated nurses that had a high QNWL had no responsibility to take care of elder parents, spouse, or special needs child (see Table 3).

## 4. Discussion

The present study aimed to assess the QNWL among nurses working in several tertiary hospitals in different regions of Saudi Arabia. The findings of this study revealed that the majority of the study participants had moderate to high QNWL scores, aligning with the study conducted in Saudi Arabia [15]. Nurses have a major contribution to health care generally, and more specifically to the patients. Therefore, measuring the nurses’ quality of life means it is necessary to know the factors that affect their quality of work and to adjust these factors in order to improve nurses’ performance.

The highest score was for the work world subscale, work context, reflecting that communication with managers, feedback, teamwork, receiving respect, and sense of security at work are some of the many work context factors that improve quality of nursing work-life. This is congruent with a previous study where 186 nurses participated and reported high work contexts due to communication, feedback, respect, and sense of belonging to the workplace as contributing factors [9]. On the other hand, the lowest score was for the work-design subscale, indicating that heavy workload with not enough time to finish the job may result in poor quality of work-life. Many other studies reported similar findings stating that nurses face heavy workload with not enough time to complete the work [11,20,21].

Overall, the study results revealed that nationality, income, being responsible for elderly parents, a spouse, or special needs child, having family accompany the nurse, and shift duration were significant factors affecting nurse quality of life. In terms of nationality, there was almost an equal percentage of nurses with high QNWL in Saudi and non-Saudi samples. Though 88.9% of nurses who scored low on QNWL were Saudi, the results are not robust enough to generalize as only 9 out of 860 participated nurses scored low QNWL, which is a small sample size to detect any difference between Saudi and non-Saudi. Additionally, there was a comparable number of nurses who achieved a moderate and high QNWL regardless of nationality. Still, the number of Saudi nurses with a moderate QNWL was almost double the number of non-Saudi nurses who had a high QNWL. This is may be a resulted from the higher percentage of Saudi nurses in the study, 61.3%. Another explanation for the Saudis being somehow dissatisfied with QNWL as compared to non-Saudi nurses could be due to the increasing family and social obligations amongst Saudi nurses, which in turn can affect the work life balance and satisfaction with QNWL.

It was expected that monthly income would be a factor associated with quality of life. However, our study finding showed that there was a negative association between salary and the quality of life. This is a surprising finding as according to the economic theory that income has a lasting impression on satisfaction as well as happiness [22]. Interestingly, Javanmardnejad et al., 2021 showed that there was no significant difference in the nurses satisfaction with their payment using multiple linear regressions [23]. On the other hand, a study showed that income was a contributing factor to emotional well-being even for nursing students [24]. This is might be due to the small number of nurses who had a low QNWL in our study, and the very high number of nurses with a moderate QNWL. Moreover, there were 333 non-Saudi nurses participating in the study, representing 38.7% of the study sample in which some of had a salary less than 10,000 SAR; however, this salary may be valued differently in their own countries, most probably worth more, and is considered as a good opportunity for them, affecting their quality of life to be moderate or high. Interestingly, having family accompany the nurse had a positive impact on nurse’s quality life work as 63.9% of the high total QNWL participating nurses had their families accompany them; *p*-value 0.001. Aligning with our study finding, a study on the factors causing depression and anxiety among health care workers in Saudi Arabia highlighted that non-Saudi health care workers being away from family reported higher depression and anxiety over their Saudi colleagues [25]. This could be due to better social support and work life balance living with family members. In term of shift duration, the length of shift was statistically significant different among those working a shorter duration compared to those working longer shifts. These findings are similar to those reported by Alharbi et al., 2019 and Venkataraman et al., 2018 [15,26]. Nurses working less hours per shift can have more time for family and social life, and this in return can lead to higher levels of satisfaction with their QNWL. Having dependent parents, a spouse, or child were associated with a higher QNWL score, as 61% of participating nurses with a high QNWL had no dependent person to take care of, giving them more time for themselves as well as for social life.

These findings could be explained by the fact that the participants in this study came from different cultural and national backgrounds. The diversity of the sample could have impacted the appreciation of factors that are perceived as motivating or demotivating by the nurses.

Conclusively, we can see that better comprehension of QNWL may lead to an in-depth understanding of nursing work life issues and ways to improve performance. Many organizations still measure job satisfaction, though research has continually demonstrated that high satisfaction does not necessarily lead to higher levels of performance or improved patient outcomes [27,28,29]. Practically, QNWL can be a better all-round measure of job satisfaction and performance. Thus, using the QNWL tool can provide comprehensive means to improve nurse’s satisfaction along with enhanced performance.

The limitations of this study included the use of a cross-sectional design and self-administered questionnaire. Therefore, further studies with more objective instruments are recommended.

## 5. Conclusions

The current study was designed to determine the level of QNWL among nurses in Saudi Arabia. The findings of this study showed that nurses in Saudi Arabia perceived a moderate to high QNWL. The demographic factors, including nationality, income, having family accompany the nurse, having an independent child, spouse, or parents, and shift duration, tend to have a statistically significant correlation with QNWL. Finally, QNWL can generate a better means to understand job satisfaction and performance over a sole understanding of job satisfaction. QNWL can enable organizations to measure the quality of nurses’ work life to unearth areas that need change, enhancement, or implement new programs designed to improve nursing work life. Thus, there is a need to identify and take the initiative to improve the areas of dissatisfaction with QNWL. This effort can provide a practical solution to better job performance and improve long-term staff nurses’ sustainability leading to better patient care.

## Figures and Tables

**Table 1:** Baseline socio-demographic characteristics (*n* = 860).

Characteristics	N (%)
Gender Males Females	62 (7.2%) 798 (92.8%)
Nationality Saudi Non-Saudi	527 (61.3%) 333 (38.7%)
Marital status Married Single Divorced Widow	619 (72.0%) 212 (24.7%) 26 (3.0%) 3 (0.3%)
Does your family accompany you in Saudi? Yes No	606 (70.5%) 254 (29.5%)
Educational level Bachelor degree High diploma degree Postgraduate diploma Master	550 (64.0%) 204 (23.7%) 65 (7.6%) 41 (4.8%)
Years of experiences <5 years 5–10 years 11–15 years >15 years	239 (27.8%) 285 (33.1%) 224 (26.0%) 112 (13.0%)
Salary in SAR <10,000 10,000 and above	422 (49.1%) 438 (50.9%)
Type of health care facility Governmental hospital Private hospital	772 (89.8%) 88 (10.2%)
Do you have any chronic medical issue? Yes No	147 (17.1%) 713 (82.9%)
Responsible for the care of your elderly parents or spouse Yes No	383 (44.5%) 477 (55.5%)
Responsible for the care of special needs children Yes No	289 (33.6%) 571 (66.4%)
Age	33.2 ± 6.1 years

**Table 2:** Quality of work life among nurses based on dimensions.

	Total Possible Score	Mean of the Total	Mean of the Total/Number of Items	Total QNWL Score Categories	Study Results N (%)
Total scale score	42–252	174.5 +/− 30.3	-	42–112 low QNWL	9 (1.0%)
				113–182 moderate QNWL	495 (57.6%)
				183–252 high QNWL	356 (41.4%)
Score for work life–home life	7–42	28	4		
Score for work design	10–60	36.2	3.62		
Score for work context	20–120	85.8	4.29		
Score for work world	5–30	19.6	3.92		

**Table 3:** The relationship between *total QNWL* and basic characteristics (*n* = 860).

Characteristics	Low Total QNWL	Moderate Total QNWL	High Total QNWL	*p*-Value
Gender Male Female	1 (11.1%) 8 (88.9%)	32 (6.5%) 463 (93.5%)	29 (8.1%) 327 (91.9%)	0.522
Nationality Saudi Non-Saudi	8 (88.9%) 1 (11.1%)	336 (67.9%) 159 (32.1%)	183 (51.4%) 173 (48.6%)	0.001
Family accompanies you in Saudi Yes No	8 (88.9%) 1 (11.1%)	371 (74.9%) 124 (25.1%)	227 (63.8%) 129 (36.2%)	0.001
Marital status Married Unmarried	7 (77.8%) 2 (22.3%)	348 (70.3%) 147 (29.7%)	264 (74.2%) 92 (25.8%)	0.432
Education Diploma and bachelor Graduate and postgraduate	7 (77.8%) 2 (22.2%)	428 (86.5%) 67 (13.5%)	317 (87.8%) 39 (12.2%)	0.240
Years of experiences ≤10 years >10 years	7 (77.8%) 2 (22.2%)	302 (61.0%) 193 (39.0%)	215 (60.4%) 141 (39.6%)	0.572
Salary in SAR ≤10,000 >10,000	2 (22.2%) 7 (77.8%)	211 (42.6%) 284 (57.4%)	209 (58.7%) 147 (41.3%)	0.001
Type of health care facility Governmental hospital Private hospital	9 (100.0%) 0 (0.0%)	448 (90.5%) 47 (9.5%)	315 (88.5%) 41 (11.5%)	0.376
Job Nature Administrative Clinical	1 (11.1%) 8 (88.9%)	70 (14.1%) 425 (85.9%)	53 (14.9%) 303 (85.1%)	0.917
Hours per shift 9.5 h 12 h	2 (22.2%) 7 (77.8%)	164 (33.1%) 331 (66.9%)	152 (42.7%) 204 (57.3%)	0.001
Chronic Medical Issue Yes No	3 (33.3%) 6 (66.7%)	91 (18.4%) 404 (81.6%)	53 (14.9%) 303 (85.1%)	0.176
Responsible for the care of special needs child, elder parents, or spouse Yes No	3 (33.3%) 6 (66.7%)	147 (29.7%) 348 (70.3%)	139 (39.0%) 217 (61.0%)	0.017

## Data Availability

The data presented in this study are available on request from the corresponding author. The data are not publicly available due to privacy.
